# Thermochemical Route for Extraction and Recycling of Critical, Strategic and High-Value Elements from By-Products and End-of-Life Materials, Part II: Processing in Presence of Halogenated Atmosphere

**DOI:** 10.3390/ma13184203

**Published:** 2020-09-21

**Authors:** Ndue Kanari, Eric Allain, Seit Shallari, Frédéric Diot, Sébastien Diliberto, Fabrice Patisson, Jacques Yvon

**Affiliations:** 1 CNRS, GeoRessources, Université de Lorraine, F-54000 Nancy, France; ericgallain@gmail.com (E.A.); frederic.diot@univ-lorraine.fr (F.D.); jyvon6355@gmail.com (J.Y.); 2 Faculty of Agriculture and Environment, Agricultural University of Tirana, 1029 Tirana, Albania; seitshallari@gmail.com; 3 CNRS, Labex DAMAS, IJL, Université de Lorraine, F-54000 Nancy, France; sebastien.diliberto@univ-lorraine.fr (S.D.); fabrice.patisson@univ-lorraine.fr (F.P.)

**Keywords:** critical and strategic materials, valuable metals, copper anode slime, PVC, de-chlorination, e-waste, thermal treatment, halogenation, metal halide volatilization

## Abstract

During the treatment of copper anode slime (CAS) under an air atmosphere, several aspects of the interactions of its main components (CuAgSe, Cu_2−x_Se_y_S_1−y_, Ag_3_AuSe_2_) with oxygen were described in Part I. As a comparative and complementary study, this work deals with the thermal behavior of CAS under air in the presence of polyvinyl chloride (PVC) between 195 and 770 °C. The preliminary thermal treatment of an e-waste sample containing brominated substances was also performed. The reaction products were systematically analyzed by scanning electron microscopy through energy-dispersive spectroscopy (SEM-EDS) and X-ray diffraction (XRD) to investigate the thermal behaviors of the studied samples in a halogenated medium. At low temperatures, the copper, silver and selenium compounds of the CAS reacted with the HCl, issued from PVC degradation, leading to the formation of their respective chlorides. Bromides of valuable metals (Cu, Pb, Sn…) were synthesized during the e-waste treatment at 500 °C and they were distributed between the solid residue and gaseous phase. The data obtained give an insight into the reactivity of several metals towards halogenated substances, which may be valuable information for conducting the extraction and recycling of targeted elements from industrial by-products and end-of-life materials by a thermochemical route.

## 1. Introduction

The results described in Part I [1] of this investigation combined with research works cited therein [2,3,4,5,6,7,8,9,10,11,12,13,14,15,16] gave a general view of needs and secondary sources for a large range of materials, identified frequently as critical and strategic materials, and they are indispensable for present and forthcoming innovations in renewable energies, transportation and cutting-edge technologies.

These materials, mostly metals, are often concentrated in the by- and/or co-products of base metals extraction and they are subsequently separated and recovered employing pyro-and/or hydrometallurgical processes. It is expected that the actual and future demand for several of these materials will grow strongly, especially to feed the production of electric and electronic devices, while the lifespans of many products such as computers and cell phones are decreasing rapidly [4]. This trend, among others, leads to the generation of a huge waste stream (electrical waste and electronic equipment, WEEE), often designated as e-waste [5,6,7,8]. Its elevated content in high added value elements (precious, strategic, critical, rare and rare earths elements), particularly for “smart parts”, makes this waste stream, a very attractive “urban mine” for the extraction of these components. But, it is necessary to emphasize that the hidden side of the e-waste stream is not all golden. These materials are composed on average of about 35% of metals, 35% of refractory oxides and ceramics and 30% of various plastics and resins. In addition to the complexity and miniaturization of the targeted metals which make their recovery difficult, the presence of chlorinated and bromated substances associated with plastics and many other components poses serious environmental problems during the recycling of e-waste. With appreciated functional properties, one of these plastics, polyvinyl chloride (PVC) is used in a broad range of products including electric and electronic appliances. With about 57% Cl (in pure PVC), it is an inherently fire-resistant plastic. However, the end of life of PVC is a real problematic case due to its high chlorine and diverse additives content. Other sources of the halogen-containing flame retardants based especially on bromine substances are widely used to meet the flammability standards of products. Several recent scientific works dealing with diverse aspects of plastics containing PVC and halogenated flame retardants are well summarized in Materials [17,18,19,20,21,22].

There are not many adequate solutions to e-waste treatment and the recycling of the contained metals. One may mention the “hot process” which resembles incineration and / or conventional pyro-metallurgical processes for the extraction of base metals (Cu, Pb…). The presence of commonly halogenated plastics complicates the operation at high temperature and causes the loss of some valuable elements. The “wet process”, meanwhile, relies on multiple steps, apparatuses and the use of a wide range of inorganic and organic reagents which are not always environmentally friendly. The use of pyrometallurgical and hydrometallurgical processes for the recovery of high-value metals from wastes was reviewed by Ding et al. [10]. Regarding smelting, it was noted that it is beneficial for the concentration of precious metals. Avarmaa et al. [11] indicated that several precious metals (gold, silver, palladium and platinum), found in end-of-life electronics, are recovered in the melted copper using a smelting process. It should be noted that high-value elements (Au, Ag, Se, Te, platinum group metals, etc.) are concentrated in the anode slime of primary copper extraction from sulfide materials. One may conclude that the processing of electronic wastes in conventional copper pyrometallurgy resulted in the concentration of the valuable elements in by-products such as the anode slime of the copper electro-refinery.

Recent studies dealt with various treatments of copper anode slime (CAS) recovering targeted elements. Among various methods used, the treatment by H_2_SO_4_-O_2_ and thiourea was given by Amer [12]. Leaching processes of the CAS using H_2_SO_4_ and HNO_3_ as digestion agents were also applied [13,14]. A combined approach, thiosulfate leaching followed by an electrodeposition, was described by Xiao et al. [15]. Use of NaOH for selenium dissolution was also developed [16]. In Part I of this investigation, the thermal behavior of CAS under an oxidizing atmosphere was described. Of special emphasis during this treatment was the recuperation of pure selenium oxide (SeO_2_) by cooling the gaseous phase during the CAS treatment.

However, limited current works are available for the thermal treatment of CAS under a dry halogenated atmosphere. In this framework, the work presented here deals with the thermal behavior of CAS supplied by a European copper plant under air in presence of polyvinyl chloride (PVC). A systematic physico-chemical characterization of the treatment products obtained under different experimental conditions has been developed in order to understand the reaction paths and mechanisms of the process for achieving a selective separation of the elements contained in the CAS. A comparison of results obtained during the treatment of CAS in solely air atmosphere with those obtained in air + PVC is often exhibited to understand the intermediate steps involved during thermochemical treatment of the CAS. Furthermore, the preliminary thermal treatment of an e-waste sample containing brominated substances was also performed. This study was performed in the frame our research program focusing mostly on the treatment of low-grade raw materials and solid residues by a thermo-chemical route [23,24,25,26,27,28] and on the valorization of certain iron bearing wastes via chemical synthesis of new and green materials [29,30,31,32,33,34].

## 2. Materials and Methods

A sample of CAS, with a median particles size lower than 63 μm, was used for this research work. Diverse analytical methods were used for the physico-chemical characterization of the CAS sample, but only the results of scanning electron microscopy–energy-dispersive spectroscopy (SEM-EDS) and X-ray diffraction (XRD) analyses are shown in this paper. The SEM-EDS and XRD equipment as well as the analytic protocol were described in Part I [1].

A sample of pure polyvinyl chloride (PVC) (without additives) was also used for this study. The e-waste sample was constituted of printed circuit boards (PCBs) obtained from end-of-life computers.

**Figure 1:**
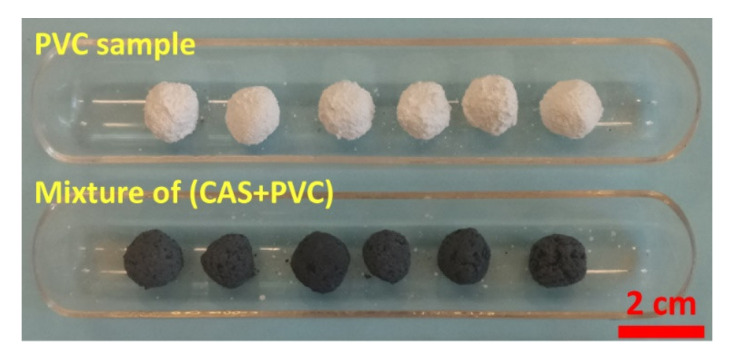
Optical image of polyvinyl chloride (PVC) and (copper anode slime (CAS + PVC) pellets used for the thermal treatment.

The PVC sample and the mixture of (CAS + PVC) sample with a mass ratio CAS/PVC = 1 were conditioned as pellets of approximately centimetric size (Figure 1) prior to thermal treatment. The PCBs were cut into strips sectioned at (10 × 2) cm without further treatment. Isothermal experimental tests for the treatment of CAS, PVC samples and mixtures of CAS with PVC (CAS + PVC) as well as for the e-waste sample were conducted in horizontal system including air metering and a static tubular furnace able to reach 1600 °C with a uniform temperature segment of at least 20 cm. The specimen holder (Figure 1) and the reactor were made of quartz material which is resistant towards the working conditions. To experiment with this process, a pre-weighted sample of several grams was introduced directly into the furnace preheated at a fixed temperature. When the dwell time is reached, the sample is removed from the furnace and cooled down to room temperature. Condensation of the outlet gases at room temperature led to the recovery of the vapor phase as condensates. Solid products (residues and condensates) were examined by SEM-EDS and XRD analysis.

## 3. Results

### 3.1. Elemental and Mineralogical Analysis of Copper Anode Slime (CAS) and Polyvinyl Chloride (PVC) Samples

**Figure 2:**
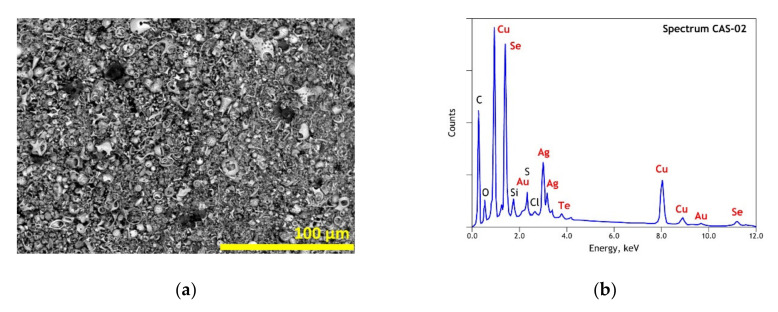
Scanning electron microscopy–energy-dispersive spectroscopy (SEM-EDS) results of initial copper anode slime: (**a**) general view (backscattered electron micrograph “BSE”) of the used sample; (**b**) overall EDS analysis of the used sample.

As noted in Part I [1], several research reports [12,13,14,15,16,35,36,37,38] emphasized the complex nature of the CAS based on their elemental, mineralogical and morphological analysis. The particular morphology of the studied CAS sample is also exhibited by an SEM image as illustrated in Figure 2a, meanwhile the multi-elemental composition (Cu, Se, Ag, S, O, Au, Si, Te, Cl) is clear in the general EDS spectrum of the CAS (Figure 2b). The presence of carbon in the EDS spectrum is probably resulting from the carbonaceous matter in the CAS sample and the carbon coating used to make the sample conductive for the SEM-EDS analysis.

**Figure 3:**
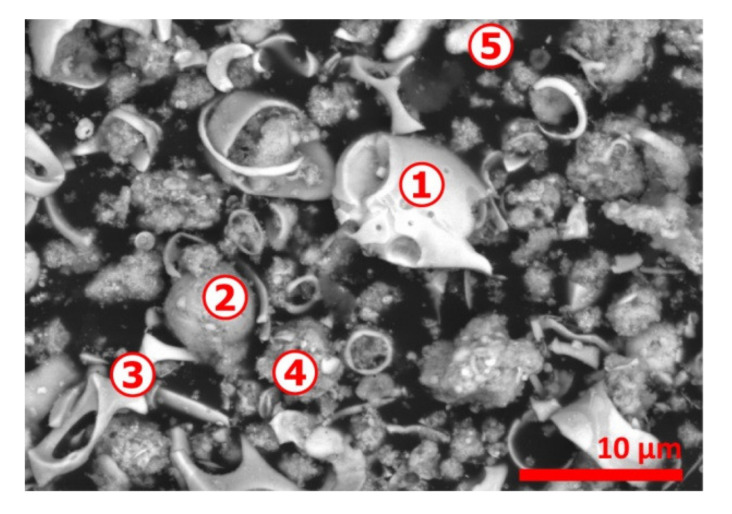
SEM-BSE image of initial copper anode slime. Numbers 1 to 5 indicate the spots for microanalysis.

A clearer view revealing the atypical morphology of the CAS sample is shown in Figure 3 and, as depicted previously [1], there are multiple particles that are irregularly and shell shaped. The SEM-EDS microanalysis data given in Table 1 indicates Cu, Se and Ag (spot n° 1, 4 and 5) are frequently associated. Sulfur and to some level tellurium are also found in the spots probed; finally, gold is identified frequently in the finest particles (spot n° 4 and 5). Several explanations for the particular state of CAS particles and their composition are provided in Part I [I] of this investigation and there are related mostly with the thermochemical reactions developing during smelting and refinery of copper.

**Figure 4:**
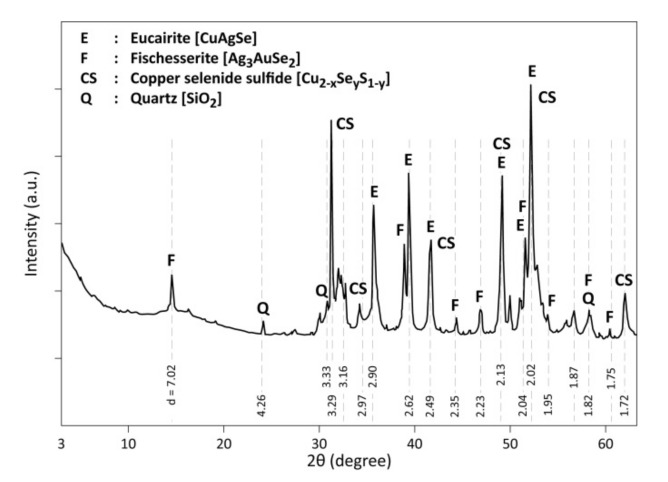
X-ray diffractogram (XRD) of copper anode sample; data adapted from [1].

The XRD diffractogram of CAS is drawn in Figure 4. It detects the presence of eucairite (CuAgSe), confirming the microanalysis performed by SEM-EDS. Furthermore, there are, in the diffractogram of CAS, some peaks corresponding to Cu_2_Se, Cu_2_S, CuSeS, as well as to non-stoichiometric compounds with an absence of certain peaks probably due to crystallite orientation and these phases are defined as copper selenide sulfide (Cu_2−x_Se_y_S_1−y_). The XRD peaks for the Au-bearing phase match mostly with fischesserite (Ag_3_AuSe_2_). This phase composition (with substitution of Ag by Cu) is also revealed by SEM-EDS analysis (spot n° 4 and 5 in Table 1). Finally, Quartz (SiO_2_) completes the list of crystallized phases identified by XRD in the CAS sample.

**Figure 5:**
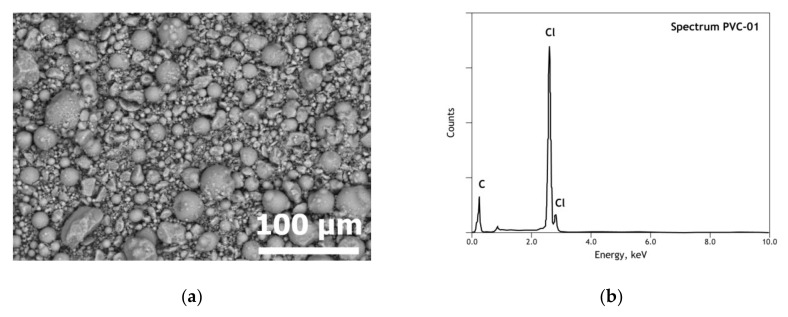
SEM-EDS results of initial PVC: (**a**) general view (BSE micrograph) of the used sample; (**b**) overall EDS analysis of the used sample.

The pure PVC sample is constituted of near-spherical particles shapes of size less than 100 μm (Figure 5a). As expected, chlorine and carbon are the only elements revealed (Figure 5b) within the element detection threshold of the SEM-EDS instrument. The XRD pattern of the used PVC sample, exhibited several diffuse halos indicating the amorphous nature of PVC.

### 3.2. Thermal Treatment of a Mixture of (CAS + PVC) and PVC Sample in Air for 1 h

**Figure 6:**
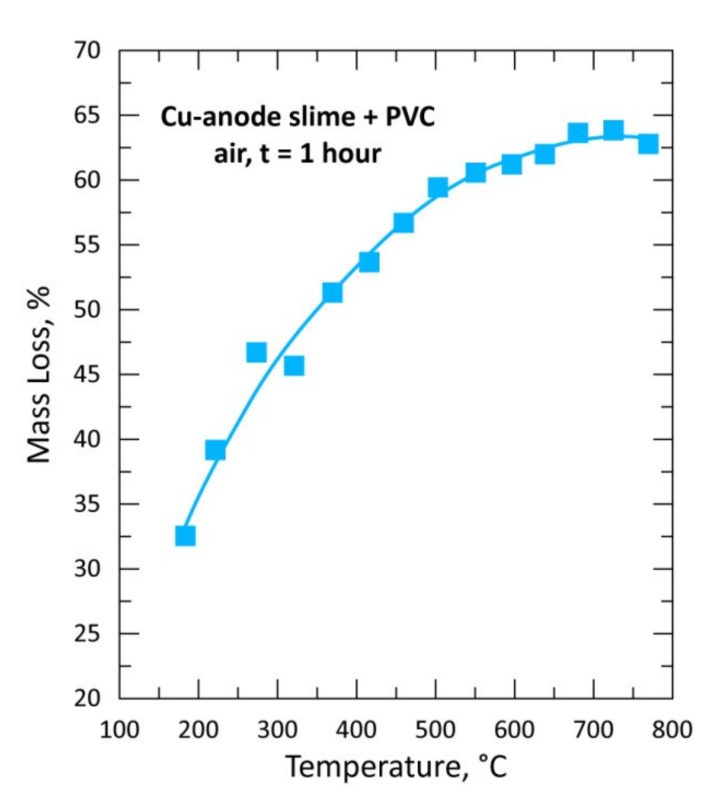
Plot of the mass loss of the sample versus temperature during treatment of CAS + PVC in air for 1 h.

**Figure 7:**
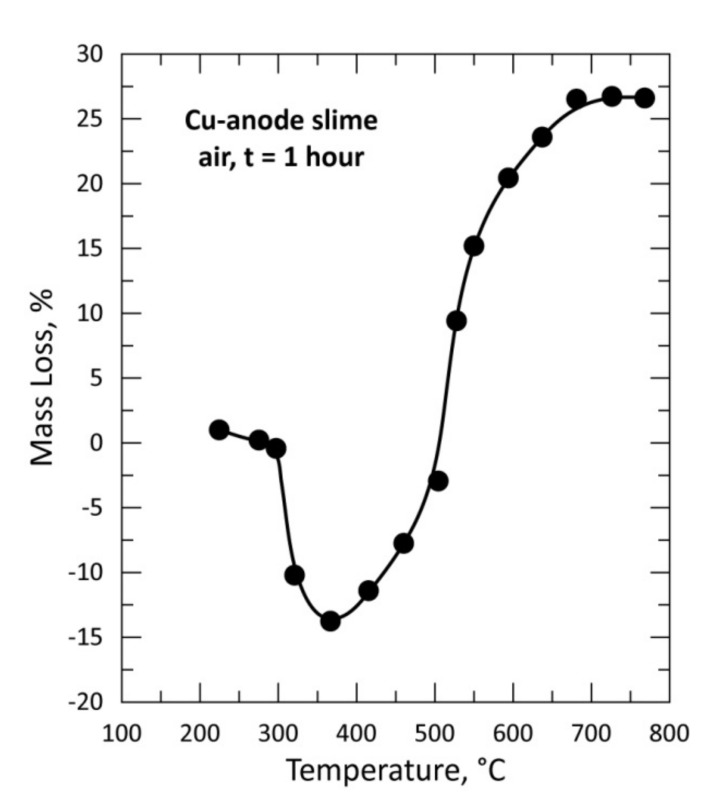
Plot of the mass loss of the sample versus temperature during treatment of CAS in air for 1 h.

Several grams of the copper anode sample mixed with PVC (CAS + PVC, CAS/PVC = 1) and conditioned as pellets (Figure 1) were used for isothermal tests performed between 195 and 770 °C in air atmosphere with a flow rate of 25 L/h. The evolution of the % mass loss (%ML) as a function of the treatment temperature is plotted in Figure 6. The %ML curve shape presents a regular continuous mass loss rise for temperatures up to about 600 °C. The examination of Figure 6 with the data obtained for the treatment of CAS alone in air atmosphere (Figure 7) shows a big difference in the curve shape, at least at low temperature. The mass gain in Figure 7 was attributed to the oxidation of several CAS elements and synthesis of combined oxides [Cu_4_O(SeO_3_)_3_, Cu_2_O(SeO_3_)], thereafter, their decomposition and subsequent volatilization of SeO_2_ led to the mass loss observed at higher temperature [1].

One option to clarify these different behaviors was to check the reaction of PVC with air at various temperatures. Additives and composition information are identified by analyzing the reaction products of the thermal treatment of (CAS + PVC) in air atmosphere, as described in Section 3.3.

**Figure 8:**
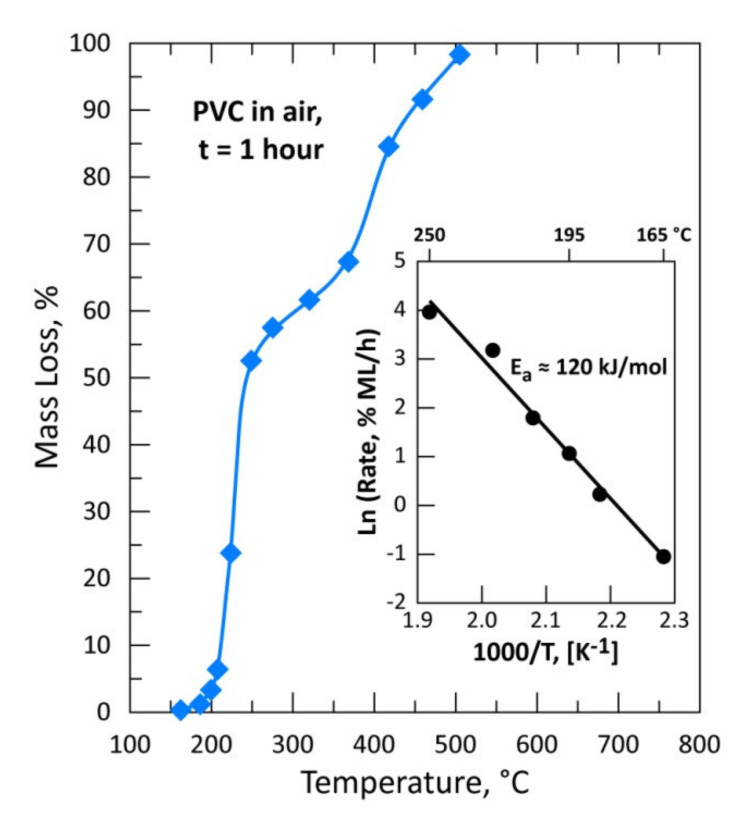
Thermal behavior of a pure PVC sample in air for 1 h.

Figure 8 is a plot of %ML of the PVC sample versus temperature ranging from 165 to 505 °C. It is clear from this figure that there are two distinguishable steps describing the thermal treatment of PVC in air atmosphere. The first step (T < 300 °C) could be attributed to a complete removal of chlorine during the thermal degradation of PVC. The value of 56.62 % ML (corresponding to the theoretical chlorine content of PVC) at 275 °C seems to indicate that all chlorine is volatilized (as HCl) leaving behind a carboniferous residue (char). The removal of chlorine is also confirmed by SEM-EDS analysis of the residue obtained at 275 °C. Other confirmation of HCl generation was also the acidity (pH) monitoring of an alkaline solution scrubbing out-gases issued from PVC treatment between 225 and 275 °C. After a latent period, the solution pH decreased abruptly from 12.5 to 0.50 as the reaction progress at 250 °C. As shown by the Arrhenius diagram in the miniaturized graphic in Figure 8, the mean apparent activation energy (E_a_) for the chlorine removal is calculated to be about 120 kJ/mol between 165 and 250 °C. The second step for the treatment of PVC in air was observed beyond 300 °C and may attributed to the reaction of the hydrocarbons with oxygen giving carbon oxides and water vapor as a final reaction product.

### 3.3. Analysis of the Reaction Products

Residues produced from isothermal processing of CAS + PVC under air atmosphere were evaluated by XRD and SEM-EDS techniques and results were compared with those obtained during the treatment of only CAS in air atmosphere.

**Figure 9:**
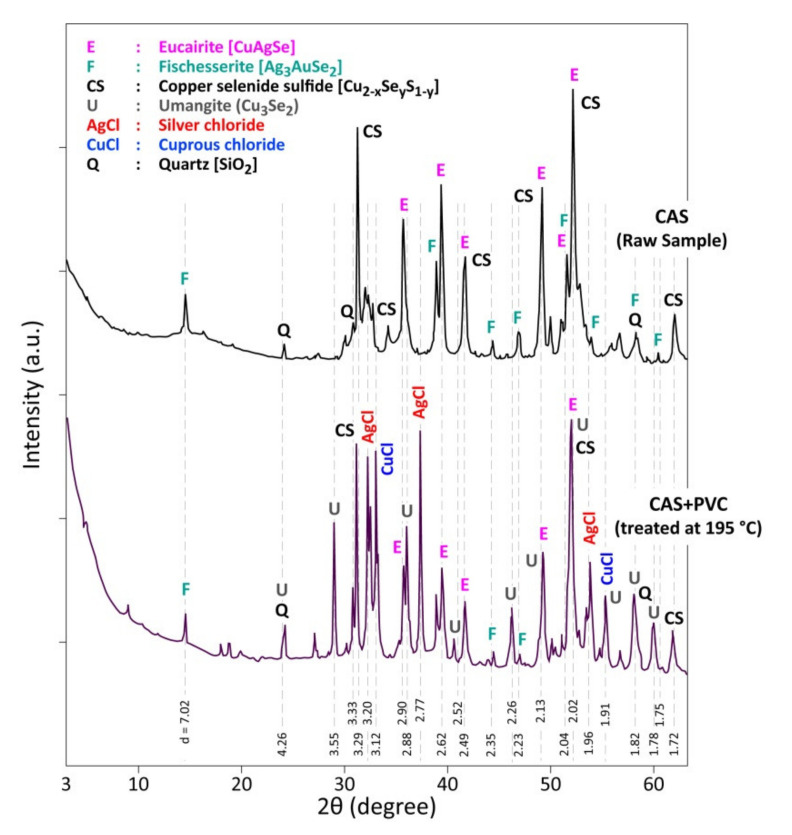
XRD patterns of a CAS raw sample and (CAS + PVC) treatment product in air at 195 °C.

Figure 9 displays the XRD patterns of the residue of CAS+PVC treated at 195 °C. Besides the characteristic peaks of the initial CAS phases (CuAgSe, Ag_3_AuSe_2_, Cu_2−x_Se_y_S_1−y_ and SiO_2_), there are new crystallized phases, namely cuprous chloride (CuCl), silver chloride (AgCl), and umangite (Cu_3_Se_2_). With this phase identification and based on thermodynamic calculations [39], one can deduce that the following overall reactions may have occurred:

1/4Cu_2_Se_(s)_ + HCl_(g)_ + 1/4O_2(g)_ → 1/2CuCl_(s)_ + 1/4SeCl_2(g)_ + 1/2H_2_O_(g)_ (1)

2Cu_2_Se_(s)_ + HCl_(g)_ + 1/4O_2(g)_ → Cu_3_Se_2(s)_ + CuCl_(s)_ + 1/2H_2_O_(g)_ (2)

1/4Ag_2_Se_(s)_ + HCl_(g)_ +1/4O_2(g)_ → 1/2AgCl_(s)_ + 1/4SeCl_2(g)_ + 1/2H_2_O_(g)_ (3)

The standard Gibbs energy changes (Δ_r_G°) at 100 °C [39] for the reaction (1), (2) and (3) are −76.41, −116.19 and −68.84 kJ/mol HCl, respectively showing the thermodynamic reactivity of the targeted phases with respect to HCl in presence of oxygen. The selenium dichloride (SeCl_2_) is considered for the thermodynamic calculation among the selenium chlorides (Se_2_Cl_2_, SeCl_2_, SeCl_4_, SeOCl_2_), although it is difficult to give an exact reactional scheme of selenium chloride formation. However, the formation of CuCl (chlorine being in the PVC) instead of CuO and the volatilization of selenium chloride (s) explain the mass loss of sample (CAS + PVC) treated at low temperature (see Figure 6). The characteristic XRD peaks of Cu_3_Se_2_ is observed in the XRD patterns of (CAS + PVC) treated at 195 °C (Figure 9). The phase diagram of the system Cu-Se [40] indicates the presence of Cu_3_Se_2_ which melted incongruently over 112 °C. One may assume that the Cu_3_Se_2_ appeared (Equation (2)) as a stable phase, perhaps during the sample cooling.

**Figure 10:**
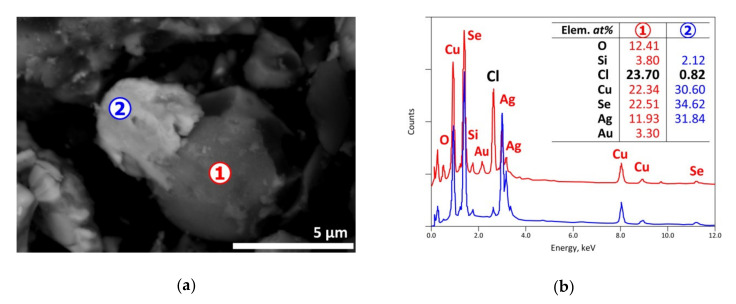
SEM-EDS results of a residue issued from the (CAS+PVC) treatment at 225 °C in air atmosphere: (**a**) general view (BSE micrograph); (**b**) EDS analysis of spot n° 1 and 2.

The presence of the partially chlorinated CuAgSe phase is observed by SEM-EDS examination for a residue obtained at 225 °C. The spot n° 2 of Figure 10 seems to indicate a small particle likely to be the CuAgSe phase. Its neighbor (spot n° 1) should be a mixture between the CuAgSe phase and Cu-Ag chlorides that are in solid state at this temperature.

**Figure 11:**
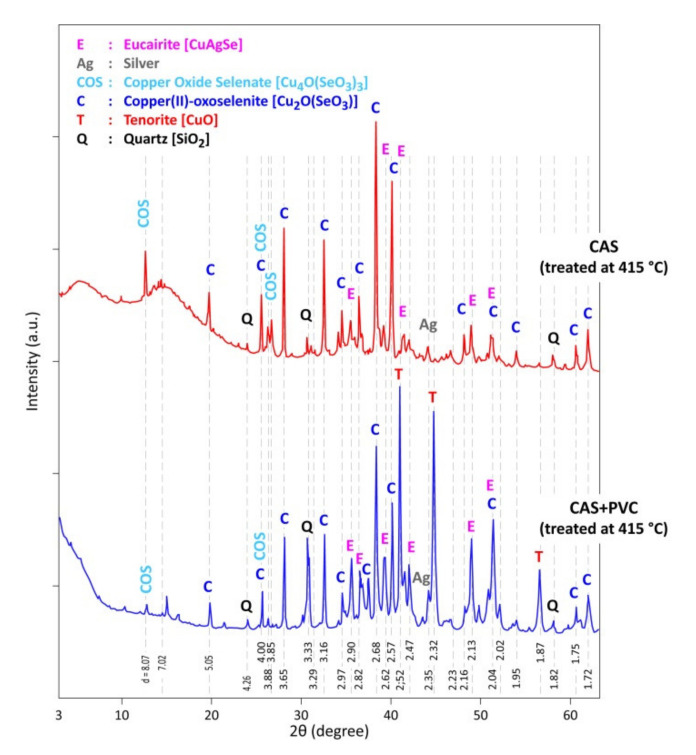
Comparison of XRD patterns of the product issued from the treatment of CAS and (CAS + PVC) samples in air at 415 °C.

The XRD results of the residues (CAS+PVC) obtained at temperatures higher than 320 °C show a good similarity with those obtained during the treatment of CAS in the absence of PVC. However, as shown in Figure 11, the decomposition of neo-formed combined oxides [e.g., Cu_4_O(SeO_3_)_3_] and the appearance of new phases [e.g., CuO], occurred apparently, at lower temperatures in the case of CAS treatment in presence of PVC. One of the plausible hypotheses is that the temperature in the reaction zone can be significantly higher than the fixed furnace temperature due to the highly exothermic nature of the reactions of the PVC degradation under oxygen. Such exothermic phenomena were also observed previously during the treatment of only 2 g of sulfides with chlorine at 300 °C resulting in a temperature increase in the reaction zone of about 30 °C [41,42].

A summary of the crystalline phases identified by XRD in the treatment residues obtained during the treatment of CAS and that of the mixture (CAS + PVC) in air for 1 h is given in Table 2 and Table 3, respectively. As shown in Table 2, the phases of the residue obtained at 225 °C are those revealed in the CAS raw sample. Eucairite (CuAgSe) is still stable at 320 °C, while the characteristic peaks of Cu_2-x_Se_y_S_1-y_ and Ag_3_AuSe_2_ phases disappeared at this temperature. The XRD of residue produced at 320 °C indicated the formation of new phases such as: [Cu_4_O(SeO_3_)_3_], [Cu_2_O(SeO_3_)]. Metallic silver is also identified in this treatment residue; note that the main XRD peaks of Ag° and Au° are overlapped. The panorama of the residues phases at 415 °C is identical of that of 320 °C, though the intensities proportion of phases changed suggesting the appearance of new phases.

At 505 °C [Cu_2_O(SeO_3_)] is still the main crystallized phase, but tenorite (CuO) is also identified and becomes the main phase at higher temperature. According to the data reported in Part I [1] and those given by Fokina et al. [43], the two steps of Cu_4_O(SeO_3_)_3_ conversion into CuO and SeO_2_ as final products can be described by Equations (4) and (5):

Cu_4_O(SeO_3_)_3_ → 2Cu_2_O(SeO_3_) + SeO_2_ (4)

2Cu_2_O(SeO_3_) → 4CuO + 2SeO_2_ (5)

Analysis of the XRD diffractograms for the residues obtained at 685 °C and 770 °C confirms the presence of well-crystallized phases of silver (Ag°), tenorite (CuO) and quartz (SiO_2_), which was present in all analyzed residues.

The reaction of HCl released during the partial degradation of the PVC with CAS at 195 °C (Table 3) led to the synthesis of new crystallized phases such as: CuCl, Cu_3_Se_2_ and AgCl which are still identified in the treatment residue obtained at 225 °C because at this temperature the rate of de-chlorination of PVC is slow and continuously producing the necessary HCl for 1 h of the experimental test (see Figure 8). At 320 °C, the phases identified in the treatment residues of CAS in the absence (Table 2) or in the presence of PVC (Table 3) are similar. One may hypothesize that starting from this temperature treatment, the kinetic of chlorine production (as HCl) from PVC accelerates, hence the contact time between CAS constituents and HCl gas is quite short. Furthermore, the used PVC is in powder form, which means that the temperature is almost uniform across the particles leading undoubtedly to the simultaneous and instantaneous de-chlorination of the PVC.

As mentioned above, a neo-formed phase such as CuO produced from thermal decomposition [Cu_2_O(SeO_3_)] via Equation (5) occurred at apparent lower temperature (at 415 °C) attributed to the heat release from constituents’ interaction in the sample mixture (CAS + PVC). Besides, the characteristic peaks of Cu_2_O (d = 2.46 Å and d = 2.13 Å) appeared also in the XRD patterns of residues obtained at 415 °C and 505 °C exhibiting the effect of the carbon on the oxidation state of copper. These diffraction peaks are of low intensity for a temperature higher than 550 °C.

**Figure 12:**
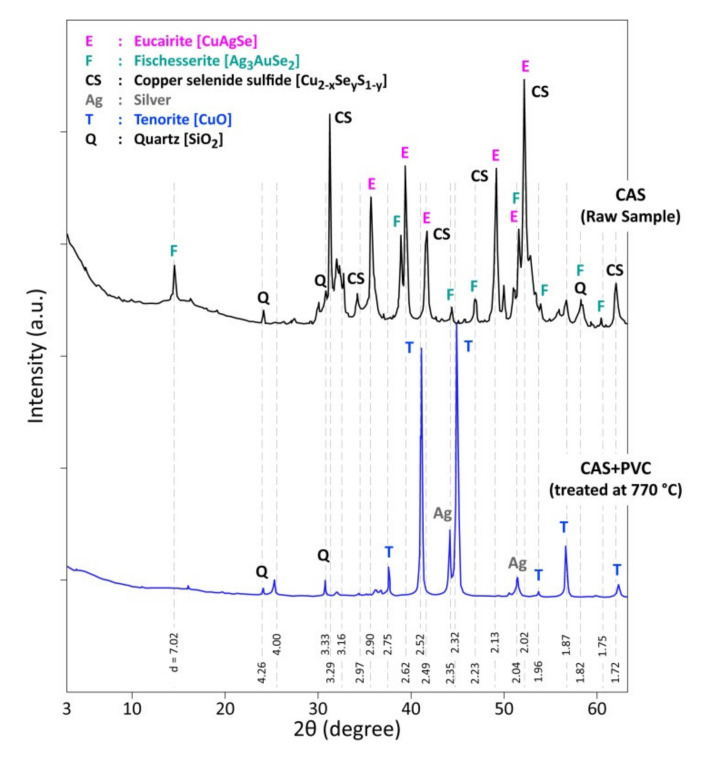
XRD diffractograms of a CAS raw sample and (CAS + PVC) thermal treatment product in air at 770 °C.

The XRD diffraction patterns of products for both CAS treatments are similar for the temperature higher than 505 °C (Table 2 and Table 3). The XRD patterns of a CAS raw sample and its reaction product issued from the thermal treatment of CAS + PVC at 770 °C are compared in Figure 12. With a complex phase composition of a raw CAS sample (CuAgSe, Cu_2−x_Se_y_S_1−y_, Ag_3_AuSe_2_ and SiO_2_), the treatment residue at 770 °C is free of the selenium-bearing phase and it is composed of CuO, SiO_2_ and Ag° (and Au°).

**Figure 13:**
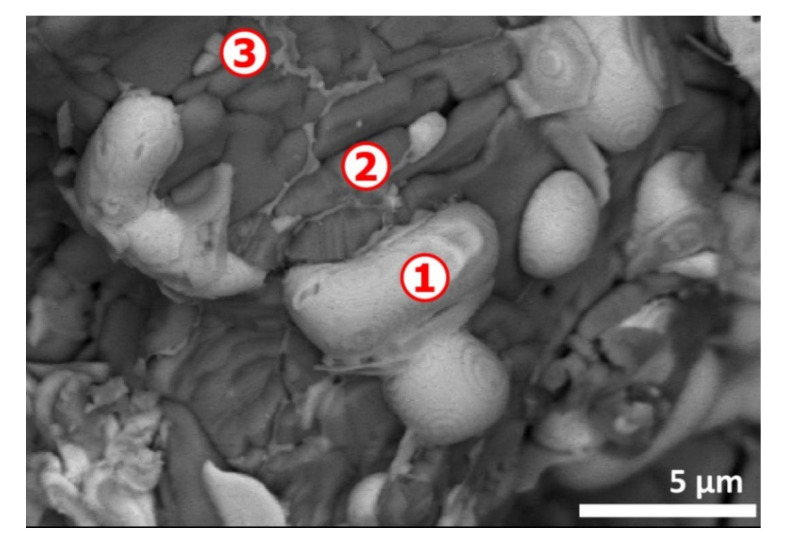
SEM aspects (BSE image) of the (CAS + PVC) sample treated at 770 °C in air atmosphere. Numbers 1 to 3 indicate the spots for microanalysis.

The EDS analysis of spot n° 1 (Figure 13) showed that Ag° (84.4 wt%) was the major constituent with some Au° (13.4 wt%) and Cu° (2.2 wt%). All smoothed particles had similar composition. The area noted by spot n° 2 was essentially composed of copper and oxygen with oxygen deficit to be CuO. The small area noted as n° 3 seemed to be composed of copper, silver and tellurium oxide (TeO_2_).

Likewise, the thermal treatment residues were examined by SEM-EDS. Figure 13 illustrates a typical morphology with a strong contrast indicating areas of distinct compositions. Data of SEM-EDX analysis of spots n° 1, 2 and 3 are reported in Table 4. The presence of well smoothed particles (image in Figure 13) indicates that the smelting of the metallic phase had been occurring during the treatment. Although the melting points of pure metals (Cu, Ag, Au) are higher than the temperature of the experimental tests, according to phase stability diagrams [40], the liquid phase appeared at temperatures lower than those of the fusion point of these metals. Furthermore, the heat release from the exothermic reactions can increase the sample temperature leading to local fusion of the treatment residue.

The chosen copper anode slime sample contained a significant amount of selenium and tellurium, which belong to the scattered elements category [44]. As reported previously [45,46,47], these elements are used in thin films (CIGS—copper indium gallium selenide, and CdTe—cadmium telluride) and used in second-generation modules of photovoltaic panels. Recovery of these elements from by-products, end-of-life solar photovoltaics and other wasted materials will be a challenge of future research works to meet the volume of industrial demand.

These results encompassed some characteristics for the thermal treatment of a copper by-product in the presence of PVC and allowed us to understand the behavior of selected element compounds during the process. However, end-of-life materials (especially electric and electronic devices) are generally more complex in elemental, chemical and mineralogical composition of inorganics, with the presence of various organic matters and additives incorporated for enhancing the functional and commodity properties of the designed appliances.

The following section gives an idea about the interaction of bromine-bearing substances with valuable metals of an e-waste (printed circuit boards—PCBs).

### 3.4. Preliminary Results for Treatment of E-Waste in Air

Several selected research works [10,11,48,49,50,51,52] were devoted to the recovery of high value added metals and other components from e-waste by using various methods. A weak point for the metal recycling from e-waste is the high amounts of halogenated substances disturbing the thermal extraction process. Of particular interest are the data reported by Hino et al. [48] and Szałatkiewicz [51] with respect to the elemental and material compositions of the PCBs originating from discarded computers. The organic epoxy resin, inorganic glass fiber and metal elements represented 31.8 wt%, 37.6 wt% and 30.1 wt% of the PCBs, respectively. The bromine content in the organic substances was 5.07 wt%.

**Figure 14:**
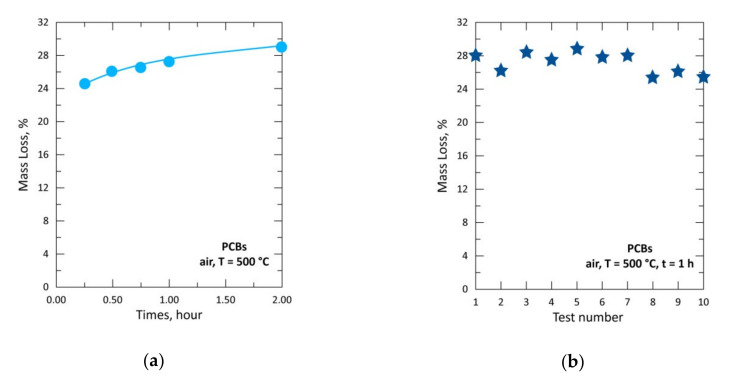
Mass change of the sample versus time for the treatment of printed circuit boards (PCBs) at 500 °C: (**a**) % mass loss (%ML) versus time; (**b**) %ML obtained for each test number at reaction time of 1 h.

In the previous sections, it has been demonstrated that hydrogen chloride issued from PVC decomposition can react with several metal compounds generating their respective chlorides. Chlorine and bromine substances are often embedded in the plastic as flame retardants and they are still widely used despite environmental concerns. To gain an insight into the thermal behavior of the e-waste (PCBs of the obsolete end-of-life computers), isothermal tests under an air atmosphere were performed at 500 °C for reaction times of 0.25, 0.50, 0.75, 1.0 and 2.0 h. As the physical sample is composed of plate strips cut at about (10 × 2) cm, it was useful to repeat the experimental tests (3 times) at a given reaction time in order to have a reliable correlation between % ML of sample and the reaction time. This allowed us to overcome the impact of macroscopic heterogeneity with respect, at least, to plastic content. Additionally, for the reaction time of 1 h, 10 experimental tests of PBCs treatment in air atmosphere were performed to carefully check the reproducibility of experimental tests and to attain a better sampling of the obtained products. All the results are displayed in Figure 14.

About 24.5% ML were obtained during the treatment of PCBs sample for 0.25 h in air atmosphere (Figure 14a). The % ML of the sample increased slightly with reaction time to reach 29.0% during the treatment at 2.0 h. The experimental tests for 1 h gave mass losses oscillating between 25.5% and 29.0% with a mean value of 27.2% (Figure 14b). The small influence the treatment time had on the sample loss is probably due to the high reactivity of the organic matter towards air (O_2_), therefore likely leading to a temperature increase in the reaction zone. The organic matter content (31.8%) of the PCBs from discarded computers previously reported [48,51] seems to be in good agreement with the results shown in Figure 14. The bromine content of the PCBs organic substances reported to be around 5 wt% in references [48,51] is assumed to apply to the similar e-waste used in the present study.

The treatment residue obtained for 1 h of treatment was sieved and the fine fraction (less than 210 μm) was analyzed by SEM-EDS technique. Likewise, the solid condensate was examined by the same technique.

**Figure 15:**
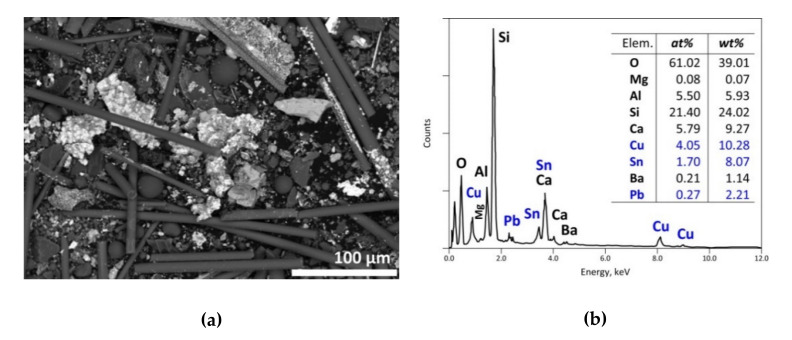
SEM-EDS results of a residue obtained from the treatment of the e-waste sample at 500 °C in air atmosphere: (**a**) general view (BSE micrograph) of the residue; (**b**) overall EDS analysis of the residue.

A general SEM image of the treatment product is displayed in Figure 15a. Although most metals (especially Cu) are found in the coarse fraction of centimetric size, this fine fraction of the treatment product still contains Sn, Cu and Pb compounds (spectrum in Figure 15b). Si, Ca, Al and Mg are often found in the sticks (see SEM image of Figure 15a) being used as reinforced fibbers and/or glass weaves for the plastics.

**Figure 16:**
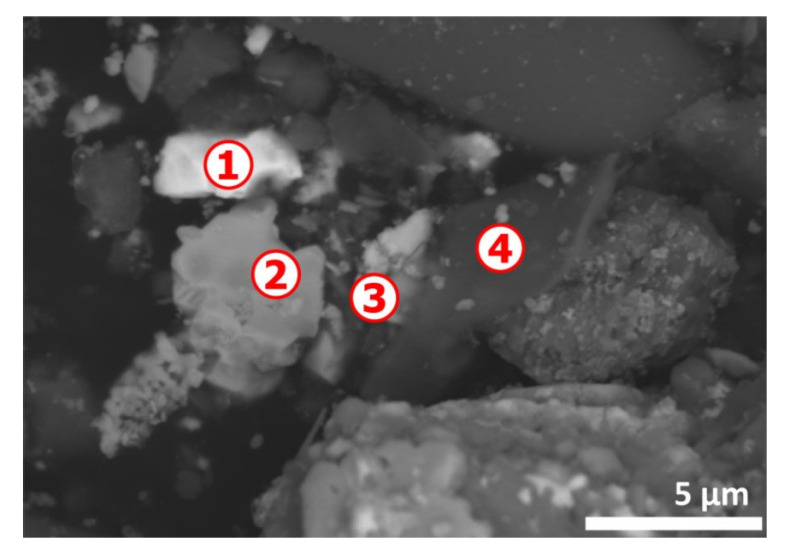
SEM aspects (BSE image) of a residue area obtained from the treatment of the e-waste sample at 500 °C in air atmosphere for 1 h. Numbers 1 to 4 indicate the spots for microanalysis.

Punctual analyses by EDS of various areas of SEM image of Figure 16 give different compositions as reported in Table 5. There are areas rich in lead (spot n° 1) and in tin (spot n° 2). Tantalum (spot n° 3) is almost certainly derived from tantalum capacitors. The EDS analyses of spot n° 4 represents mostly the elemental composition of an inorganic filler particle.

**Figure 17:**
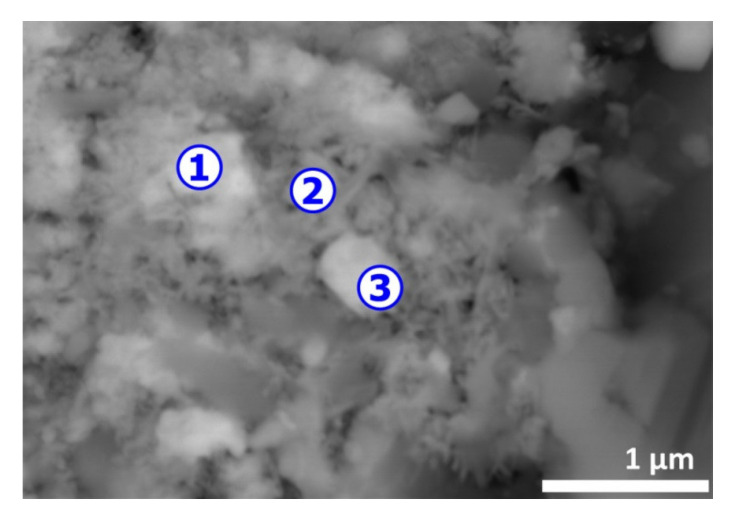
SEM aspects (BSE image) of a residue area obtained from the treatment of the e-waste sample at 500 °C in air atmosphere for 1 h. Numbers 1 to 3 indicate the spots for microanalysis.

The attractive result of the SEM-EDS analysis is given in Figure 17 and Table 6. Besides metals (Ag, Cu, Pb, Sn…), at various proportions, there is an appreciable amount of bromine (spots n° 1 to 3) indicating that bromine is bonded with part of the metals as bromides.

**Figure 18:**
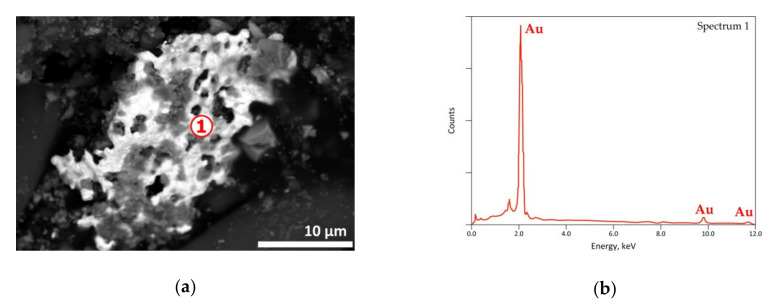
SEM-EDS results of a residue obtained from the treatment of the e-waste sample at 500 °C in air atmosphere: (**a**) general view (BSE micrograph) of a residue area; (**b**) punctual EDS spectrum.

As shown in Figure 18, gold, which is used in PCBs to assure circuit board contacts, seems to be inert to the treatment at this temperature.

A thermodynamic calculation [39] showed that the values of the Δ_r_G° for the reaction (6), (7), (8) and (9) at 500 °C are −119.80, −124.11, −143.22 and –119.97 kJ/mol HBr, respectively. Such negative values suggest that the envisaged reactions are favorable from the thermodynamic point of view. The species AgBr, CuBr, PbBr_2_ and SnBr_4_ are chosen for their predominant thermodynamic stability in the studied system (Metal-O-Br) and based on the thermochemical data availability.

Ag_(s)_ + HBr_(g)_ + 1/4O_2(g)_ → AgBr_(l)_ + 1/2H_2_O_(g)_ (6)

Cu_(s)_ + HBr_(g)_ + 1/4O_2(g)_ → CuBr_(l)_ + 1/2H_2_O_(g)_ (7)

1/2Pb_(s)_ + HBr_(g)_ + 1/4O_2(g)_ → 1/2PbBr_2(l)_ + 1/2H_2_O_(g)_ (8)

1/4Sn_(s)_ + HBr_(g)_ + 1/4O_2(g)_ → 1/4SnBr_4(g)_ + 1/2H_2_O_(g)_ (9)

**Figure 19:**
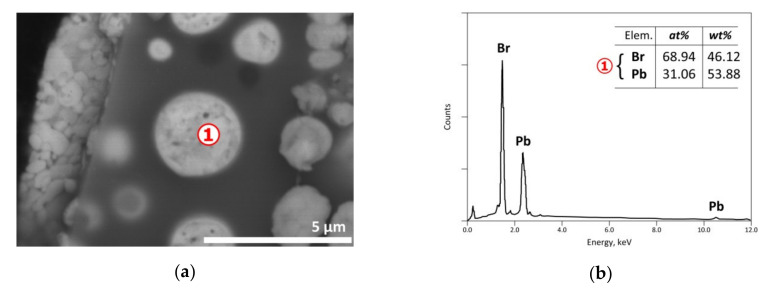
SEM-EDS results of a condensate issued from the e-waste sample treated at 500 °C in air atmosphere: (**a**) general view (BSE micrograph); (**b**) EDS analysis of spot n° 1.

**Figure 20:**
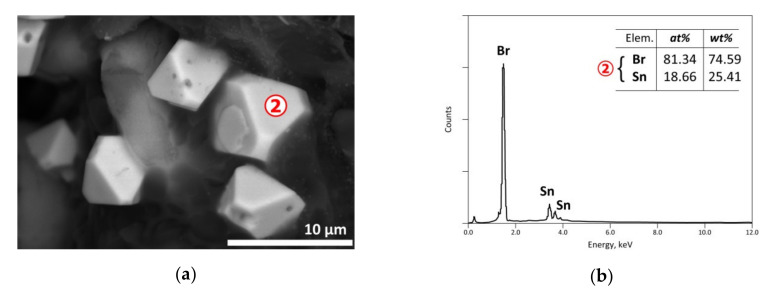
SEM-EDS results of a condensate issued from the e-waste sample treated at 500 °C in air atmosphere: (**a**) general view (BSE micrograph); (**b**) EDS analysis of spot n° 2.

As indicated in Section 2, the exit gases were cooled resulting in the condensation of the vapor phase and the recovery of a solid condensate mixed often with unburnt carbonaceous matter, soot and liquid giving the visual appearance of a pasty mass. SEM-EDS results of a condensate are shown in Figure 19 with an EDS analysis of spot n° 1 corresponding roughly to PbBr_2_. As the vapor pressure of PbBr_2_ is near to 0.1 kPa [39], its presence in the condensate is probably due to it being brought back by the carrier gas and/or thanks to temperature increase enhancing its volatilization. Another EDS punctual analysis displayed in Figure 20b, noted as spot n° 2 of SEM image, suggested most likely the stannic bromide (SnBr_4_) generated from the condensation of gaseous tin bromide [SnBr_4(g)_] in the cooled part of the reactor. The obtained results showed that several metals could be concentrated in the vapor phase via volatilization of the neo-formed halides during the thermal process.

As revealed during this study, the extraction of the critical and high-value elements from the chosen by-product and end-of-life materials represents a challenge due to the chemical and mineralogical complexity of their components as well as to the selectivity of the extractive chemistry of different metals for an efficient separation. Moreover, fair thermodynamic data, the ability to gather phases differentiation, punctual information about elemental content, in addition to monitoring the morphological and textural evolution of the thermally treated samples, are all contributing to a better understanding of the reaction mechanism involved and processing steps for the studied cases. Other aspects of this investigation, dealing with mass balance of the processes and assessment of technical and economical evaluation, should be carried out in future studies.

## 4. Conclusions

Copper Anode Slime (CAS) is a complex material containing the targeted elements (Ag, Au, Se, Te, Cu…) distributed in diverse mineralogical phases such as Ag_3_AuSe_2_, CuAgSe, Cu_2−x_Se_y_S_1−y_.

The interaction of CAS constituents with HCl, issued from the degradation of polyvinyl chloride (PVC) under in an air atmosphere, started from about 200 °C leading to the favorable formation of AgCl and CuCl. Selenium is probably volatilized as selenium chlorides, most likely as SeCl_2(g)_.

The isothermal degradation of PVC in an air atmosphere is a two-step process. The PVC de-chlorination occurs at temperature lower than 300 °C and proceeds with an apparent activation energy of around 120 kJ/mol. The second step should be attributed to the reaction of oxygen with carbonaceous matter generated in the first step. Full sample degradation and volatilization of the reaction products were achieved during treatment of PVC at about 500 °C for 1 h.

The reaction of HCl generated from PVC with CAS at temperature higher than 275 °C remained limited because the generation rate of HCl was higher than that of the interaction of HCl with CAS constituents.

The thermal treatment of CAS + PVC beyond 300 °C gave roughly similar results to the processing of CAS alone in air atmosphere. The formation of combined copper-selenium oxides, followed by their decomposition allowing solid copper oxides are the main steps of this treatment.

The final residue of the CAS + PVC treatment in air at temperature higher than 600 °C is composed of CuO (Cu_2_O) and alloys of Ag-Au (with some Cu°), while tellurium is found as oxide (TeO_2_), and silica (SiO_2_) which is unreactive with respect to the thermal treatment. Further treatment of this product is required for the final separation of these high-value element compounds.

The bromine-bearing flame retardants of the printed circuits boards (PCBs) also appeared to be a good halogenating agent for several components of this e-waste. Bromides of copper, lead and tin, synthetized through the reaction of the evolved HBr with their respective metals, are distributed between residue and condensate of the PCBs treatment at 500 °C under air atmosphere. Further studies linked with various experimental parameters should be undertaken for a selective separation of the synthetized halides.

## Figures and Tables

**Table 1:** Elemental composition of copper anode slime analyzed by SEM-EDS.

Elements	Spot *n*° 1		Spot *n*° 2		Spot *n*° 3		Spot *n*° 4		Spot *n*° 5	
	^1^wt%	^1^at%	wt%	at%	wt%	at%	wt%	at%	wt%	at%
O	-	-	-	-	-	-	1.75	8.15	3.49	15.70
Si	-	-	-	-	-	-	0.31	0.82	0.49	1.25
S	1.17	2.87	3.65	7.84	2.05	4.44	3.30	7.66	2.06	4.62
Cu	32.40	40.13	47.16	51.11	48.48	53.10	32.60	38.22	28.05	31.73
Se	33.15	33.05	41.97	36.60	44.68	39.37	28.99	27.34	23.06	20.99
Ag	30.27	22.09	5.50	3.51	4.79	3.09	17.01	11.74	33.38	22.24
Te	3.01	1.86	1.71	0.92	-	-	-	-	-	-
Au	-	-	-	-			16.04	6.07	9.47	3.46

^1^ wt% and at% represent mass and atomic percentage, respectively.

**Table 2:** Summary of XRD results of the residues issued from the treatment of CAS in air for 1 h.

Identified Phases	IS ^1^	225 °C	320 °C	415 °C	505 °C	685 °C	770 °C
CuAgSe							
Ag_3_AuSe_2_							
Cu_2−x_Se_y_S_1−y_							
Silver (Ag)							
Cu_4_O(SeO_3_)_3_							
Cu_2_O(SeO_3_)							
CuO							
SiO_2_							

^1^ Initial Sample.

**Table 3:** Summary of XRD results of the residues issued from the treatment of the mixture (CAS + PVC) in air for 1 h at various temperatures.

Identified Phases	IS ^1^	195 °C	225 °C	320 °C	415 °C	505 °C	685 °C	770 °C
CuAgSe								
Ag_3_AuSe_2_								
Cu_2−x_Se_y_S_1−y_								
Cu_3_Se_2_								
CuCl								
Silver (Ag)								
AgCl								
Cu_4_O(SeO_3_)_3_								
Cu_2_O(SeO_3_)								
CuO								
SiO_2_								

^1^ Initial sample.

**Table 4:** Elemental composition (EDS data) of the (CAS + PVC) treated at 770°C in air atmosphere.

Elements	Spot n° 1		Spot n° 2		Spot n° 3	
	^1^wt%	^1^at%	wt%	at%	wt%	at%
O	-	-	12.85	37.29	4.38	17.85
Al	-	-	-	-	0.14	0.35
Si	-	-	-	-	0.11	0.26
Cu	2.16	3.84	83.92	61.32	60.52	62.04
Ag	84.40	88.45	3.23	1.39	18.40	11.11
Au	13.44	7.71	-	-	-	-
Te	-	-	-	-	16.45	8.40

^1^ wt% and at% represents mass and atomic percentage, respectively.

**Table 5:** Elemental composition (EDS data) of the areas displayed in Figure 16.

Elements	Spot n° 1		Spot n° 2		Spot n° 3		Spot n° 4	
	^1^wt%	^1^at%	wt%	at%	wt%	at%	wt%	at%
O	1.89	15.62	21.39	65.63	20.75	66.29	46.96	66.27
Na	-	-	-	-	-	-	3.21	3.16
Al	-	-	0.65	1.19	1.64	3.11	11.92	9.97
Si	0.30	1.40	0.70	1.22	-	-	17.41	14.00
P	-	-	-	-	-	-	2.12	1.54
Ca	1.81	5.99	-	-	-	-	3.37	1.90
Ti	-	-	-	-	-	-	2.09	0.98
Mn	-	-	-	-	12.83	11.94	-	-
Cu	5.79	12.06	-	-	-	-	1.47	0.52
Br	7.13	11.82	-	-	-	-	-	-
Sn	-	-	77.26	31.96	2.38	1.02	5.02	0.95
Ta	-	-	-	-	62.40	17.63	-	-
Pb	83.08	53.11	-	-	-	-	6.43	0.70

^1^ wt% and at% represent mass and atomic percentage, respectively.

**Table 6:** Elemental composition (EDS data) of the areas displayed in Figure 17.

Elements	Spot n° 1		Spot n° 2		Spot n° 3	
	^1^wt%	^1^at%	wt%	at%	wt%	at%
O	17.75	60.63	14.56	54.58	16.91	58.83
Cu	4.42	3.80	4.95	4.67	8.53	7.47
Br	9.88	6.75	16.52	12.39	16.84	11.73
Ag	4.39	2.22	9.73	5.41	2.57	1.32
Sn	49.28	22.69	33.07	16.71	28.60	13.41
W	4.18	1.24	2.99	0.98	2.92	0.88
Pb	10.11	2.67	18.17	5.26	23.64	6.35

^1^ wt% and at% represents mass and atomic percentage, respectively.
